# Serum small extracellular vesicle–associated snoRNA panel adds diagnostic value to CEA and CYFRA21–1 for non-small cell lung cancer: an exploratory single-center study

**DOI:** 10.3389/fonc.2026.1881438

**Published:** 2026-09-11

**Authors:** Liguang Wang, Mei Wang, Qun Zhang, Xinyi Li, Zhijun Zhang, Kangyu Wang

**Affiliations:** 1 Tumor Research and Treatment Center, Shandong Provincial Hospital Affiliated to Shandong First Medical University, Jinan, Shandong, China; 2 Department of Pharmacy, Shandong Provincial Hospital Affiliated to Shandong First Medical University, Jinan, Shandong, China; 3 Department of Blood Transfusion, The Affiliated Taian City Central Hospital of Qingdao University, Taian, China; 4 Department of Clinical Laboratory Medicine, The First Affiliated Hospital of Shandong First Medical University and Shandong Provincial Qianfoshan Hospital, Jinan, Shandong, China; 5 Department of Clinical Laboratory, The Affiliated Taian City Central Hospital of Qingdao University, Taian, China; 6 Shandong Provincial Key Medical and Health Laboratory of Anti-drug Resistant Drug Research, Taian City Central Hospital, Taian, China

**Keywords:** diagnostic modeling, internal validation, liquid biopsy, non-small cell lung cancer, serum tumor markers, small extracellular vesicles, small nucleolar RNA, snoRNA

## Abstract

**Background:**

Circulating small extracellular vesicle (sEV)-associated small nucleolar RNAs (snoRNAs) have emerged as candidate biomarkers because of their stability in biofluids. However, whether these molecular signals provide diagnostic information beyond established serum tumor markers remains uncertain. This study evaluated a four-snoRNA panel and investigated its incremental value when combined with carcinoembryonic antigen (CEA) and cytokeratin-19 fragment (CYFRA21-1) for NSCLC discrimination.

**Methods:**

This retrospective single-center case-control study included 240 Untreated patients with pathologically or cytologically confirmed NSCLC and 205 healthy controls. A discovery microarray was performed using RNA isolated from the sEV-enriched fraction of three NSCLC samples and three controls. Four candidate snoRNAs (AC007956.1, AC093523.1, AL358875.1, and SNORA51) were subsequently quantified by quantitative reverse-transcription PCR using ΔCt values. Logistic regression models were developed for the four-snoRNA panel, the clinical marker model (CEA plus CYFRA21-1), and the combined six-marker model. Model performance was assessed using repeated stratified 10-fold cross-validation (20 repetitions), training-fold-derived thresholds, 2,000 stratified bootstrap resamples, calibration metrics, paired DeLong comparisons, subgroup analyses, and age/sex-matched sensitivity analyses.

**Results:**

The discovery dataset contained 936 analyzable snoRNA probes. Although 134 probes showed nominal differential expression (*P* < 0.05), none remained significant after false-discovery-rate correction. NSCLC cases were older than controls (62.5 vs. 42.3 years; standardized mean difference, 1.73), whereas sex distributions were comparable. The four-snoRNA model achieved a cross-validated AUC of 0.924 (95% CI, 0.898-0.948), compared with 0.903 (95% CI, 0.874-0.931) for the CEA plus CYFRA21–1 model. The combined six-marker model showed the highest discrimination, with an AUC of 0.970 (95% CI, 0.955-0.983), sensitivity of 87.1%, specificity of 96.6%, calibration intercept of -0.018, calibration slope of 0.923, and Brier score of 0.064. Compared with the clinical model, the six-marker model increased AUC by 0.067 (95% CI, 0.043-0.092; paired DeLong *P* < 0.001). Model performance remained consistent in stage 0-II disease (AUC, 0.963; 95% CI, 0.940-0.980) and age/sex-matched analyses (AUC, 0.964; 95% CI, 0.941-0.983). No snoRNA marker retained significant associations with clinical characteristics after multiple-testing correction.

**Conclusions:**

A serum sEV-associated four-snoRNA panel showed incremental diagnostic value when combined with CEA and CYFRA21–1 in this exploratory NSCLC case-control study. The resulting six-marker model maintained discrimination during repeated internal cross-validation, including analyses restricted to early-stage disease and age/sex-matched cohorts.

## 1 Introduction

Lung cancer remains the leading cause of cancer-related mortality worldwide, with non-small cell lung cancer (NSCLC) accounting for approximately 85% of all cases (1, 2). Low-dose computed tomography (LDCT) screening has reduced lung cancer mortality among high-risk populations and has been incorporated into screening programs in several countries (3–5). However, LDCT frequently detects indeterminate pulmonary nodules, many of which are ultimately benign. This limitation creates a clinical need for additional biomarkers that can improve risk stratification and assist decision-making while reducing unnecessary invasive procedures (6).

Liquid biopsy provides minimally invasive access to tumor-associated molecular signals circulating in blood, including circulating tumor DNA (ctDNA), circulating tumor cells, extracellular vesicles (EVs), and extracellular RNA species (7, 8). ctDNA analysis can capture tumor-specific genomic alterations and evolutionary changes, but its diagnostic sensitivity remains limited in early-stage disease because of the low abundance of tumor-derived DNA in circulation (9). EV-associated RNA molecules have attracted increasing interest because the lipid bilayer structure of EVs protects encapsulated RNA cargo from extracellular degradation, enabling detection in biofluids under relatively stable conditions. Small nucleolar RNAs (snoRNAs) are conserved non-coding RNAs primarily involved in ribosomal RNA modification, ribosome biogenesis, and related regulatory processes (10, 11).

Aberrant snoRNA expression has been reported in lung cancer tissues, circulating specimens, and tumor-associated cellular models, indicating that snoRNAs may reflect cancer-related molecular alterations. Liao et al. identified distinct snoRNA expression patterns associated with NSCLC diagnosis and prognosis (12), whereas Mei et al. demonstrated the oncogenic function of SNORA42 in lung cancer progression (13). Mannoor et al. characterized snoRNA signatures associated with tumor-initiating cell properties (14), and Gao et al. profiled global snoRNA alterations in lung cancer using whole-genome sequencing approaches (15). More recently, Cui et al. reported functional and prognostic associations involving NOP10-related snoRNAs in lung cancer cohorts and experimental models (16).Despite these observations, most previous studies have focused on tissue expression profiles, mechanistic regulation, or tumor-associated cellular phenotypes. The clinical diagnostic value of circulating snoRNAs, particularly their incremental contribution beyond established serum biomarkers, remains insufficiently characterized. Biological association alone does not establish clinical utility; candidate biomarkers require evaluation in clinically relevant cohorts using integrated diagnostic models. Recent studies have extended this concept to extracellular vesicle (EV)-associated snoRNAs and suggested that serum EV-associated snoRNA signatures may distinguish malignant from benign pulmonary nodules (17). These findings support further investigation of EV-associated snoRNAs as circulating diagnostic candidates. However, whether EV-associated snoRNAs can provide additional diagnostic information beyond routinely measured serum tumor markers, such as CEA and CYFRA21-1, remains unclear. CEA and CYFRA21–1 are commonly used serum biomarkers in NSCLC evaluation, although their sensitivity is limited when applied individually (18). Determining whether EV-associated snoRNAs provide complementary information to these conventional markers is essential for assessing their potential clinical relevance.

In this exploratory study, we evaluated a serum small-EV-associated four-snoRNA panel comprising AC007956.1, AC093523.1, AL358875.1, and SNORA51 in patients with NSCLC. We first examined candidate snoRNA signals identified by small RNA microarray screening and subsequently quantified selected markers using quantitative reverse-transcription PCR. We then assessed the diagnostic performance of snoRNA-based, clinical marker-based, and combined models using repeated internal cross-validation. Additional analyses examined model performance in early-stage disease and age/sex-matched cohorts.

The objective of this study was not to establish a clinically validated diagnostic assay, but to determine whether EV-associated snoRNA signals could provide incremental diagnostic information beyond conventional serum markers and to generate evidence for future validation studies.

## 2 Materials and methods

### 2.1 Study design and reporting framework

This retrospective, single-center case-control study was conducted at the Affiliated Tai’an City Central Hospital of Qingdao University between July and December 2023. The study was reported according to the Transparent Reporting of a Multivariable Prediction Model for Individual Prognosis or Diagnosis (TRIPOD) statement and the Standards for Reporting Diagnostic Accuracy Studies (STARD 2015) recommendations (19–21).The study flow diagram summarizes the participants included in the final analytical cohort. Model development and internal evaluation were performed within the available cohort using repeated internal cross-validation. No independent external validation or prospective validation cohort was available.

### 2.2 Participants and reference diagnosis

Patients with NSCLC were consecutively available cases who underwent serum collection before initiation of anticancer treatment. NSCLC diagnosis was confirmed by pathological or cytological examination. Patients with previous anticancer therapy were excluded. Additional exclusion criteria included other active malignancies, severe active infection, active autoimmune disease, and severe organ dysfunction. Tumor stage was classified according to the eighth edition of the International Association for the Study of Lung Cancer (IASLC) TNM staging system (22).The final case cohort comprised 240 patients, including 187 adenocarcinomas and 53 squamous cell carcinomas. Stage distribution was as follows: stage 0 (n=23), stage I (n=80), stage II (n=17), stage III (n=56), and stage IV (n=64).Healthy controls were recruited from the hospital health-examination center. Available exclusion criteria included known pulmonary disease, acute or chronic inflammatory conditions, recent surgery, and other malignancies.

### 2.3 Serum processing and small-EV enrichment

Peripheral blood samples were collected before anticancer treatment. Serum was separated by centrifugation at 2,000 ×g for 10 min, followed by clarification at 12,000 ×g for 10 min at 4 °C. Samples were stored at -80 °C until analysis. For small-EV enrichment, serum samples were centrifuged at 10,000 ×g for 30 min at 4 °C. The supernatant was subsequently ultracentrifuged at 100,000 ×g for 2 h at 4 °C using a Beckman Coulter ultracentrifuge. The resulting pellets were washed with phosphate-buffered saline and subjected to a second ultracentrifugation step under identical conditions. The final preparation was defined as a small-EV-enriched fraction based on vesicular morphology, particle-size distribution, and detection of EV-associated proteins. Transmission electron microscopy was performed using 15 μL of the EV-enriched preparation. Samples were fixed with 1% glutaraldehyde, negatively stained, and examined using a Tecnai G2 Spirit microscope. Particle size and concentration were assessed by nanoparticle-tracking analysis using a ZetaView PMX110 system. EV-associated proteins CD9, CD63, and CD81 were detected by western blotting.

### 2.4 Discovery microarray

Total RNA was extracted from the small-EV-enriched fraction of three NSCLC samples and three healthy controls using TRIzol reagent. RNA quantity and integrity were assessed using NanoDrop and Bioanalyzer or denaturing gel electrophoresis. Small RNA expression profiling was performed using the Arraystar Human Small RNA Microarray platform. Samples underwent dephosphorylation, terminal labeling, hybridization, and scanning using an Agilent G2505C microarray scanner. Signal intensities were log2 transformed and quantile normalized. A total of 936 analyzable snoRNA probes were included in the discovery dataset. Differential expression analysis was initially performed using nominal P values and fold-change criteria for candidate prioritization. Multiple-testing correction was performed using the Benjamini–Hochberg procedure, with an adjusted q value <0.05 considered statistically significant.

### 2.5 qRT-PCR and conventional serum markers

RNA was extracted from the small-EV-enriched fraction using 500 μL TRIzol reagent. Reverse transcription was performed using the Mix-X miRNA First-Strand Synthesis Kit (Accurate Biotechnology). Quantitative PCR was performed on a LightCycler 480 system (Roche) using SYBR Green chemistry. Each reaction contained 2.0 μL cDNA, 6.4 μL RNase-free water, 0.8 μL forward primer, 0.8 μL reverse primer, and 10.0 μL SYBR Green master mix in a final volume of 20 μL.U6 was used as the endogenous reference. Relative expression was calculated using ΔCt values defined as Ct(target) − Ct(U6), with lower ΔCt values indicating higher relative abundance. Primer sequences are provided in **Table 1**.

**Table 1:** qRT-PCR primer sequences.

snoRNA	Forward primer (5imerd	Reverse primer (5imere
AC007956.1	TACTTCCAACTCTAGGATAGCATCA	TCAAGCTGGGAACGGAATGG
AC093523.1	CCCTTACCTGTTTCTCCTTTGC	TTAATCACGTTTCATGCATCTCCA
AL358875.1	GCTCTTGGCACTGGTTTGCT	TTGCATTCTCTTCCCCTCACTC
SNORA51	GGCCTCCTGGTGCTTACC	TCACAATACAGACCAGGCACA

Serum CEA and CYFRA21–1 concentrations were measured using electrochemiluminescence immunoassay on a Cobas e602 analyzer (Roche Diagnostics).

### 2.6 Statistical analysis and model development

The primary outcome was discrimination between patients with NSCLC and healthy controls. Continuous variables were summarized as mean ± standard deviation or median (interquartile range), according to data distribution. Age differences were assessed using Welch’s t test, sex distributions using Pearson’s chi-square test, and biomarker distributions using the Mann-Whitney U test. Standardized mean differences were calculated to quantify baseline imbalance. False-discovery-rate correction was applied to discovery-array analyses, biomarker comparisons, and case-only association analyses using the Benjamini–Hochberg procedure. Seven prespecified logistic regression models were developed:(1) demographic model (age and sex);(2) four-snoRNA model;(3) clinical model (log-transformed CEA and CYFRA21-1);(4) six-marker model (four snoRNAs combined with CEA and CYFRA21-1);(5) demographic plus four-snoRNA model;(6) demographic plus clinical model; (7) demographic plus six-marker model. No automated variable selection procedures were applied.

Model performance was estimated using repeated stratified 10-fold cross-validation with 20 repetitions. Model coefficients and classification thresholds were derived exclusively from training folds and applied to corresponding held-out folds. Out-of-fold predicted probabilities were aggregated across repetitions for discrimination and calibration assessment. The area under the receiver operating characteristic curve (AUC), sensitivity, specificity, calibration intercept, calibration slope, and Brier score were estimated with 95% confidence intervals derived from 2,000 stratified bootstrap resamples. Correlated ROC curves were compared using DeLong’s test. Exploratory decision-curve analysis was performed. Prespecified subgroup analyses included stage 0-I disease, stage 0-II disease, stage I disease, adenocarcinoma, and squamous cell carcinoma. An age- and sex-matched sensitivity analysis was performed using exact sex matching. All statistical tests were two-sided, and *P* values <0.05 were considered statistically significant. Primary analyses were performed using Python 3.11.

## 3 Results

### 3.1 Cohort flow and data completeness

**Figure 1:**
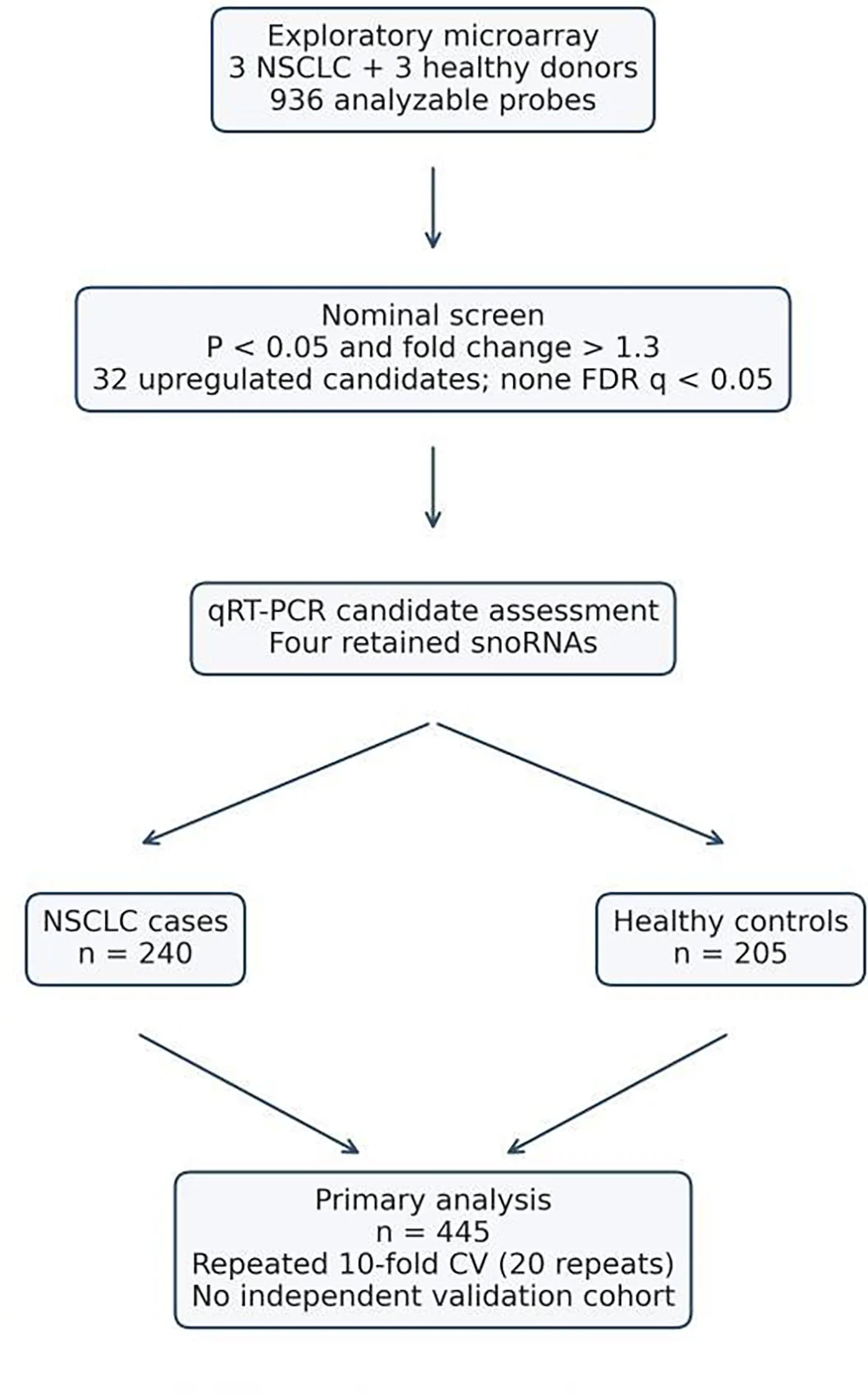
Study flow and final analytic cohorts.

The analytical dataset consisted of a discovery microarray cohort (three NSCLC cases and three healthy controls) and a qRT-PCR cohort including 240 NSCLC cases and 205 healthy controls (**Figure 1**). After data integration, no duplicate identifiers were identified. Complete information on outcome status, demographic characteristics, serum tumor markers (CEA and CYFRA21-1), and the four selected snoRNA candidates was available for all participants. Smoking information was unavailable for healthy controls.

### 3.2 Characterization of the serum small-EV-enriched fraction and discovery screen

**Figure 2:**
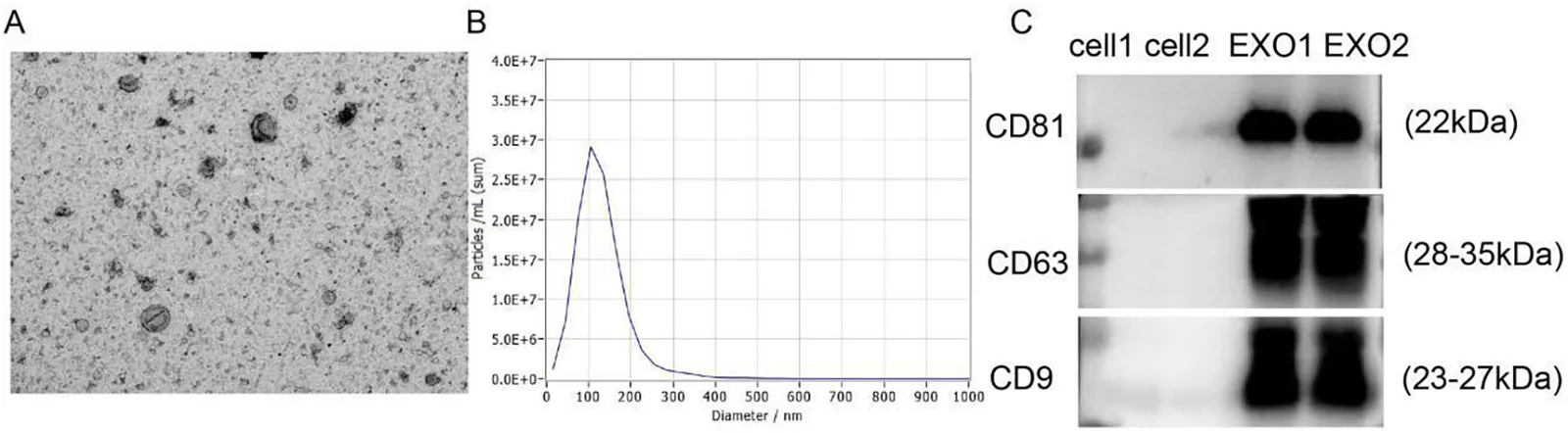
Characterization of the serum small-EV-enriched fraction. **(A)** Representative transmission electron microscopy. **(B)** Nanoparticle-tracking size distribution. **(C)** Western blot detection of CD81, CD63, and CD9 in EV-enriched preparations. Positive findings support EV enrichment.

**Figure 3:**
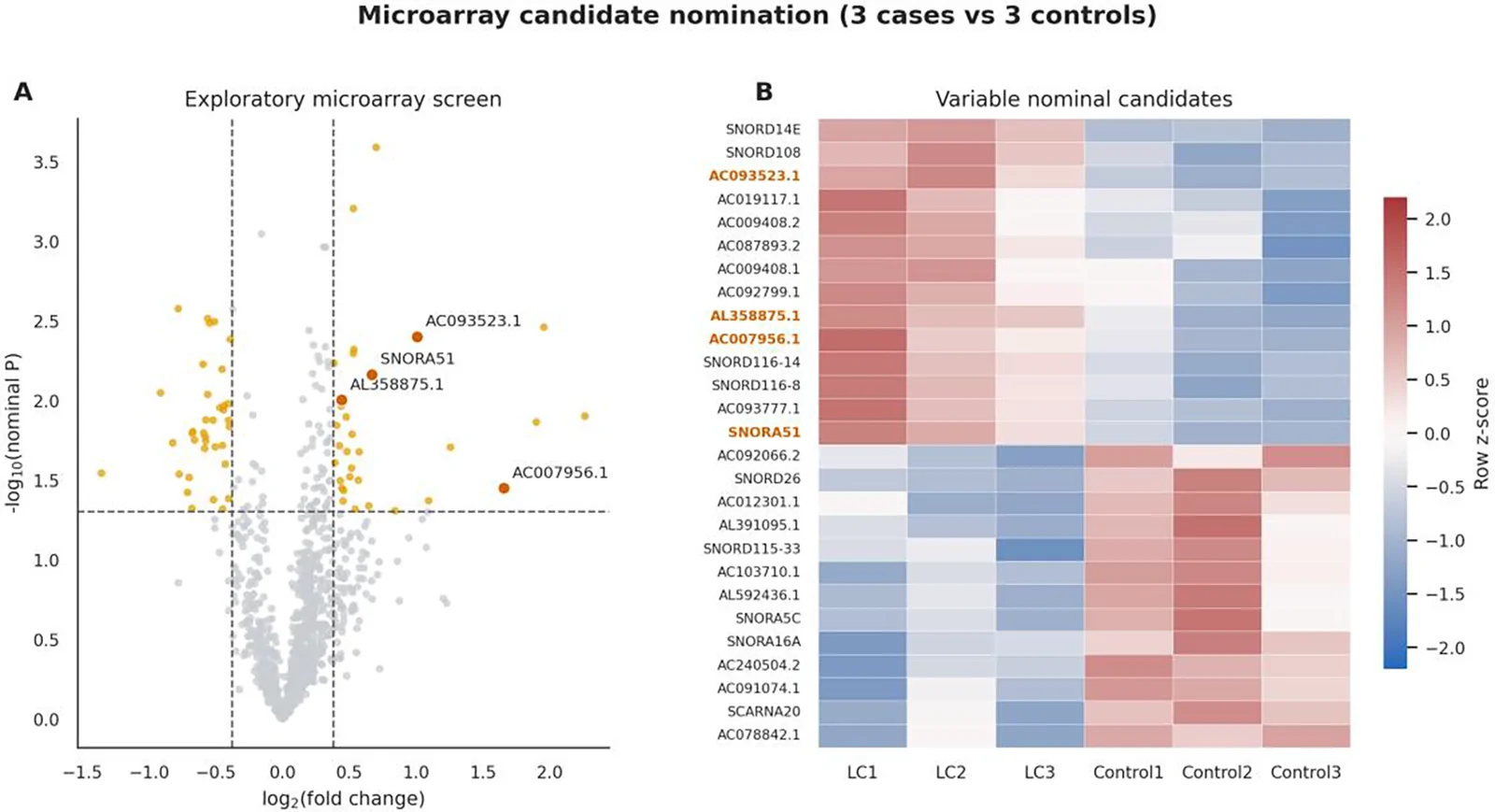
Exploratory microarray results in three NSCLC cases and three healthy controls. The volcano plot shows fold change and nominal P values for 936 transcripts; the heatmap displays the selected candidates.

Transmission electron microscopy demonstrated rounded vesicular structures within the isolated fraction. Nanoparticle-tracking analysis showed particle-size distributions consistent with a small-EV-enriched preparation. Western blotting confirmed the presence of EV-associated markers CD9, CD63, and CD81 (**Figure 2**).The discovery microarray contained 936 analyzable snoRNA probes. Among these, 134 probes showed nominal differential expression (*P* < 0.05), and 32 demonstrated nominal upregulation with fold change >1.3. None of the detected probes remained significant after Benjamini–Hochberg correction (q<0.05), with the lowest observed q value of 0.204 (**Figure 3**).Four candidate snoRNAs were selected for subsequent qRT-PCR quantification based on prespecified exploratory criteria, including effect magnitude, nominal statistical significance, and assay feasibility. The microarray-derived characteristics of the selected candidates were as follows: AC093523.1 (fold change, 2.01; *P* = 0.004; q=0.238), SNORA51 (fold change, 1.59; *P* = 0.007; q=0.238), AL358875.1 (fold change, 1.36; *P* = 0.009; q=0.262), and AC007956.1 (fold change, 3.15; *P* = 0.035; q=0.321).

### 3.3 Participant characteristics and biomarker distributions

**Figure 4:**
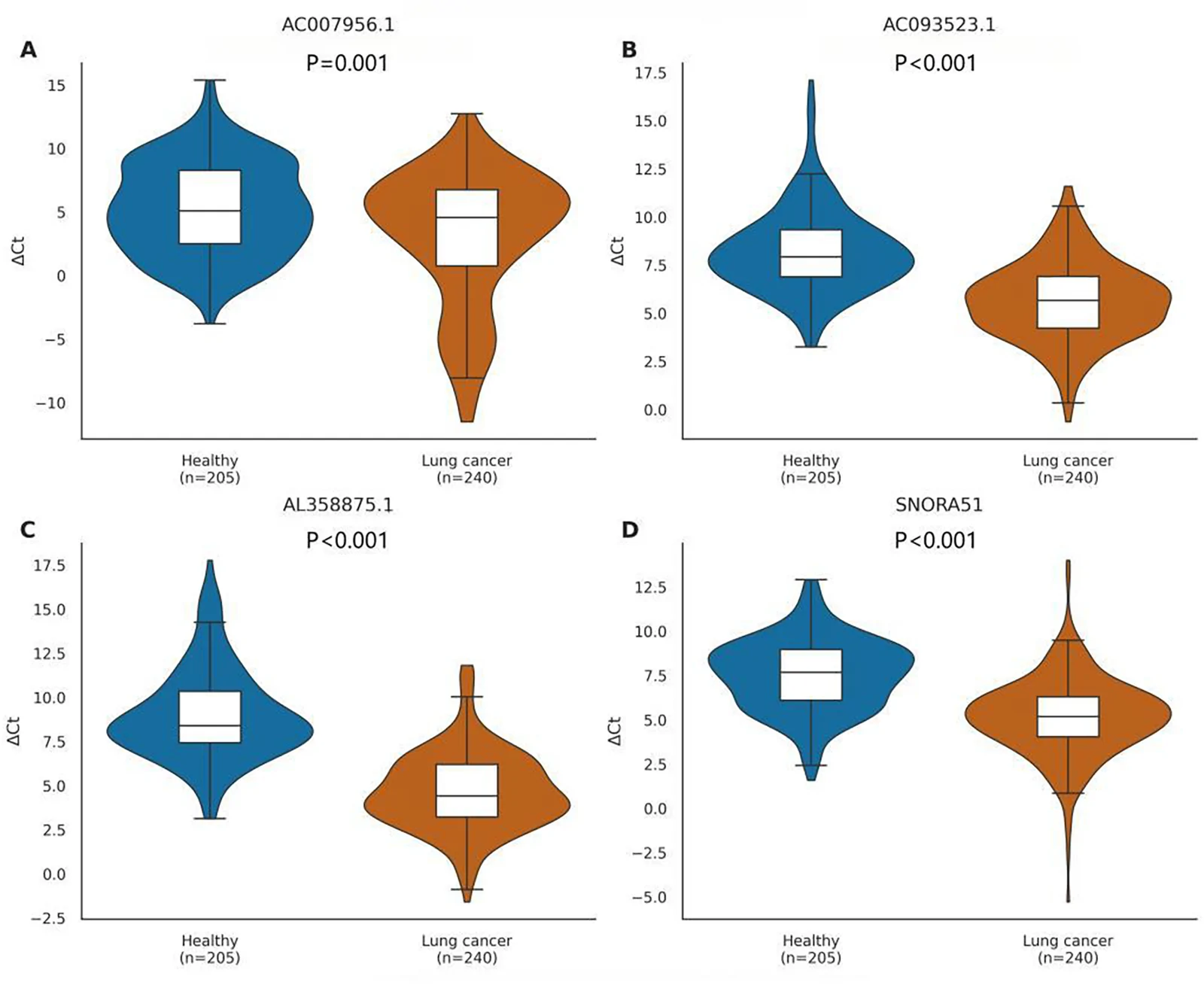
qRT_PCR ΔCt value distributions of AC007956.1 **(A)**, AC093523.1 **(B)**, AL358875.1 **(C)**, and SNORA51 **(D)** in 240 NSCLC patients and 205 healthy controls.

NSCLC cases were older than healthy controls (mean age, 62.51 vs. 42.31 years; *P* < 0.001), with substantial baseline imbalance indicated by the standardized mean difference (SMD = 1.73). Sex distribution was comparable between groups (male proportion, 56.2% vs. 52.7%; *P* = 0.511).Serum concentrations of CEA and CYFRA21–1 were higher in NSCLC cases than in controls. All four candidate snoRNAs showed lower ΔCt values in NSCLC samples, indicating higher relative abundance compared with healthy controls (**Table 2**; **Figure 4**).

**Table 2:** Baseline characteristics of NSCLC cases and healthy controls.

Variable	NSCLC (n=240)	Healthy controls (n=205)	Effect/SMD	P_value	Test	FDR_q
Age, years	62.51 ± 10.55	42.31 ± 12.70	1.730	<0.001	Welch t test	
Male sex, n (%)	135 (56.2)	108 (52.7)	0.072	0.511	Pearson chi-square	
CEA(ng/mL)	3.37 (2.12-9.47)	1.55 (1.04-2.20)	0.641	<0.001	Mann-Whitney U	<0.001
CYFRA21_1(ng/mL)	3.84 (2.61-7.60)	1.97 (1.51-2.46)	0.690	<0.001	Mann-Whitney U	<0.001
AC007956.1(ΔCt)	4.57 (0.75-6.76)	5.09 (2.50-8.28)	-0.190	0.001	Mann-Whitney U	0.001
AC093523.1(ΔCt)	5.66 (4.22-6.91)	7.93 (6.88-9.34)	-0.639	<0.001	Mann-Whitney U	<0.001
AL358875.1(ΔCt)	4.41 (3.22-6.20)	8.40 (7.42-10.35)	-0.827	<0.001	Mann-Whitney U	<0.001
SNORA51(ΔCt)	5.18 (4.03-6.30)	7.68 (6.09-8.96)	-0.595	<0.001	Mann-Whitney U	<0.001

SMD, standardized mean difference; IQR, interquartile range.

### 3.4 Repeated cross-validation performance of diagnostic models

**Figure 5:**
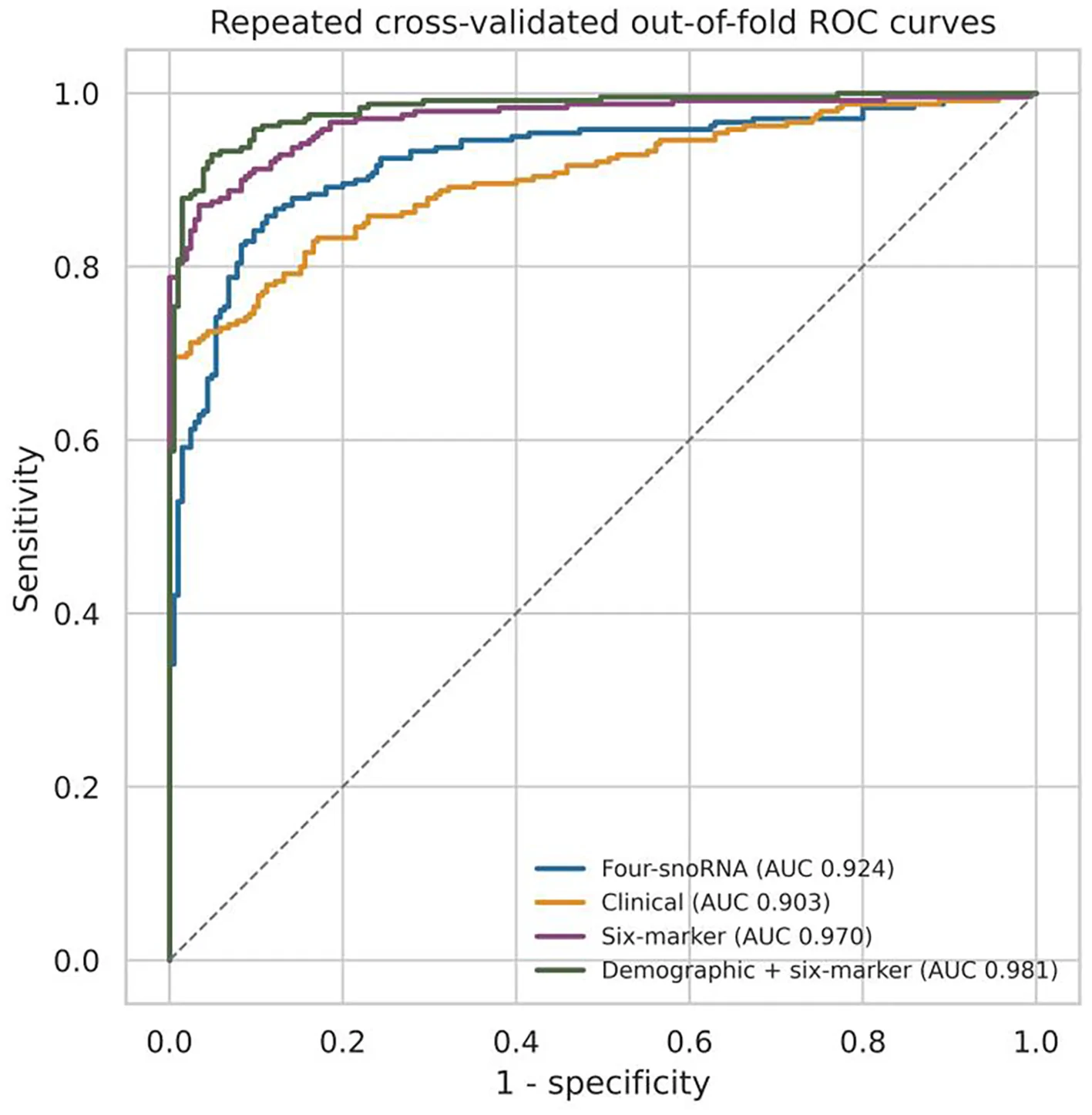
Receiver operating characteristic curves from patient-level averaged out-of-fold probabilities over 20 repeats of stratified 10-fold cross-validation.

Across 20 repetitions of stratified 10-fold cross-validation, the four-snoRNA model achieved a mean cross-validated AUC of 0.924 (95% CI, 0.898-0.948). The corresponding sensitivity, specificity, calibration intercept, calibration slope, and Brier score were 85.8%, 88.8%, −0.004, 0.961, and 0.105, respectively. The clinical model incorporating CEA and CYFRA21–1 achieved an AUC of 0.903 (95% CI, 0.874-0.931), with sensitivity of 69.6%, specificity of 99.5%, and a Brier score of 0.120.The combined six-marker model showed the highest discrimination, with an AUC of 0.970 (95% CI, 0.955-0.983), sensitivity of 87.1%, specificity of 96.6%, calibration intercept of -0.018, calibration slope of 0.923, and Brier score of 0.064.The demographic plus six-marker model achieved an AUC of 0.981 (**Table 3**; **Figure 5**).In paired ROC comparisons, the six-marker model showed higher discrimination than the clinical model (ΔAUC=0.067; 95% CI, 0.043-0.092; *P* < 0.001) and the four-snoRNA model (ΔAUC=0.046; 95% CI, 0.026-0.067; *P* < 0.001).Adding snoRNA markers to the demographic plus clinical model resulted in a modest improvement in discrimination (ΔAUC=0.023; 95% CI, 0.009–0.038; P = 0.002, **Table 4**).

**Table 3:** Repeated cross-validated performance of logistic models.

Model	AUC	AUC_low	AUC_high	Sensitivity	Specificity	Calibration_intercept	Calibration_slope	Brier
Demographic	0.887	0.855	0.916	0.863	0.756	0.004	0.979	0.133
Four-snoRNA	0.924	0.898	0.948	0.858	0.888	-0.004	0.961	0.105
Clinical	0.903	0.874	0.931	0.696	0.995	-0.002	0.976	0.120
Six-marker	0.970	0.955	0.983	0.871	0.966	-0.018	0.923	0.064
Demographic + four-snoRNA	0.961	0.943	0.977	0.929	0.898	0.003	0.938	0.072
Demographic + clinical	0.958	0.941	0.974	0.883	0.922	-0.001	0.954	0.078
Demographic + six-marker	0.981	0.970	0.991	0.929	0.951	-0.017	0.863	0.049

**Table 4:** Paired DeLong comparisons using out-of-fold predictions.

Model_1	Model_2	Delta_AUC	CI_low	CI_high	DeLong_P
Six-marker	Clinical	0.067	0.043	0.092	<0.001
Six-marker	Four-snoRNA	0.046	0.026	0.067	<0.001
Demographic + six-marker	Demographic + clinical	0.023	0.009	0.038	0.002
Demographic + six-marker	Demographic	0.095	0.066	0.124	<0.001

### 3.5 Calibration, decision analysis, subgroup performance, and sensitivity analyses

**Figure 6:**
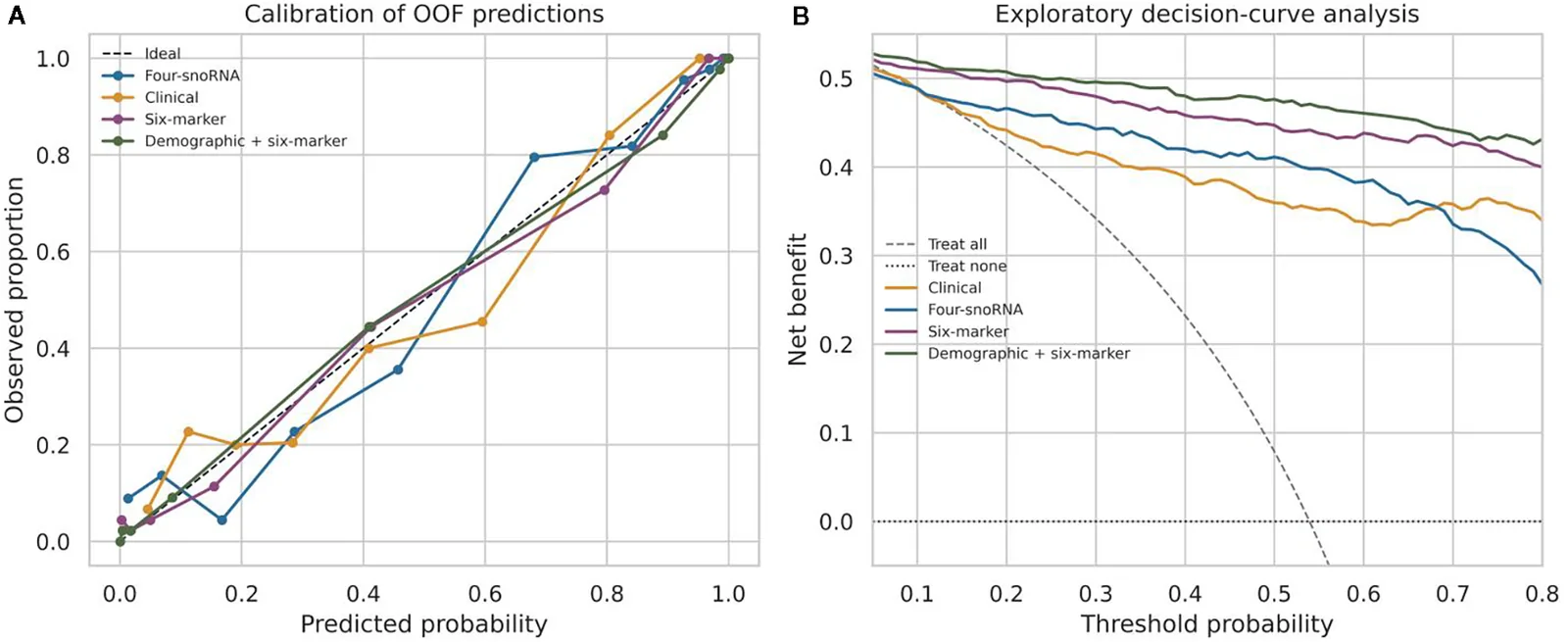
Calibration and exploratory decision-curve analysis. **(A)** Calibration of averaged out-of-fold probabilities. **(B)** Net benefit across probability thresholds.

Calibration assessment based on averaged out-of-fold predictions showed good agreement between predicted and observed risks for the primary models (**Figure 6A**). The six-marker model demonstrated a calibration intercept of -0.018 and a calibration slope of 0.923, indicating limited model optimism. Exploratory decision-curve analysis showed higher relative net benefit estimates for the six-marker model compared with the clinical model across the evaluated threshold range (**Figure 6B**). Because the case-control design generated an artificial outcome prevalence, these findings were interpreted as comparative model behavior rather than direct estimates of population-level clinical utility. The six-marker model retained strong discrimination in early-stage disease, with AUCs of 0.959 for stage 0-I disease, 0.963 for stage 0-II disease, and 0.971 for stage I disease (**Table 5**).

**Table 5:** Exploratory subgroup performance.

Subgroup	Cases_n	Controls_n	Model	AUC	CI_low	CI_high
All NSCLC	240	205	Four-snoRNA	0.924	0.898	0.948
All NSCLC	240	205	Clinical	0.903	0.874	0.931
All NSCLC	240	205	Six-marker	0.970	0.955	0.983
All NSCLC	240	205	Demographic + six-marker	0.981	0.970	0.991
Stage 0-I	103	205	Four-snoRNA	0.928	0.893	0.957
Stage 0-I	103	205	Clinical	0.844	0.792	0.891
Stage 0-I	103	205	Six-marker	0.959	0.934	0.978
Stage 0-I	103	205	Demographic + six-marker	0.975	0.957	0.988
Stage 0-II	120	205	Four-snoRNA	0.932	0.898	0.961
Stage 0-II	120	205	Clinical	0.854	0.803	0.898
Stage 0-II	120	205	Six-marker	0.963	0.940	0.980
Stage 0-II	120	205	Demographic + six-marker	0.978	0.963	0.990
Stage I only	80	205	Four-snoRNA	0.955	0.932	0.974
Stage I only	80	205	Clinical	0.847	0.790	0.901
Stage I only	80	205	Six-marker	0.971	0.952	0.986
Stage I only	80	205	Demographic + six-marker	0.982	0.968	0.993
Adenocarcinoma	187	205	Four-snoRNA	0.917	0.885	0.945
Adenocarcinoma	187	205	Clinical	0.885	0.849	0.917
Adenocarcinoma	187	205	Six-marker	0.963	0.942	0.979
Adenocarcinoma	187	205	Demographic + six-marker	0.977	0.961	0.989
Squamous cell carcinoma	53	205	Four-snoRNA	0.949	0.907	0.979
Squamous cell carcinoma	53	205	Clinical	0.965	0.933	0.990
Squamous cell carcinoma	53	205	Six-marker	0.995	0.986	1.000
Squamous cell carcinoma	53	205	Demographic + six-marker	0.998	0.995	1.000

Across histological subgroups, the six-marker model achieved AUCs of 0.963 for adenocarcinoma and 0.995 for squamous cell carcinoma. The estimate for squamous cell carcinoma should be interpreted cautiously because of the limited sample size (n=53). In the age- and sex-matched sensitivity analysis including 108 matched case-control pairs, the six-marker model maintained robust discrimination, achieving an AUC of 0.964 (95% CI, 0.941-0.983), compared with 0.915 for the four-snoRNA model and 0.884 for the clinical model.

### 3.6 Case-only association analyses

No statistically significant associations were observed between individual snoRNA markers and clinical characteristics, including age, sex, smoking status, histological subtype, and tumor stage, after Benjamini–Hochberg correction.AC007956.1 showed nominal associations with male sex (β=−2.81; *P* < 0.05) and ordinal tumor stage (β=0.48 per stage increment; *P* < 0.05). Neither association remained significant after multiple-testing correction (q=0.258 and 0.447, respectively).

## 4 Discussion

In this exploratory single-center case-control study, we evaluated whether serum small-EV-associated snoRNA signals could provide additional diagnostic information beyond conventional serum biomarkers for NSCLC. The combined six-marker model incorporating four snoRNAs with CEA and CYFRA21–1 achieved higher discrimination than the clinical marker model alone, with an internally cross-validated AUC of 0.970 compared with 0.903 for CEA and CYFRA21-1. The improvement in discrimination was accompanied by increased sensitivity (87.1% vs. 69.6%) with a modest reduction in specificity (96.6% vs. 99.5%). The model also showed favorable internal calibration, with a calibration intercept of -0.018, a calibration slope of 0.923, and a Brier score of 0.064.These findings indicate that EV-associated snoRNA measurements may provide additional discriminatory information when combined with established serum markers.

Previous studies have reported altered snoRNA expression patterns in lung cancer tissues, circulating specimens, and tumor-associated cellular models (16, 17, 23, 24). These investigations have linked snoRNA dysregulation to processes including ribosome biogenesis, RNA modification, tumor-cell proliferation, and tumor-associated phenotypes. Other studies have further suggested that snoRNAs may reflect biological differences related to tumor progression and patient outcomes, indicating that these molecules may have functions beyond their traditional use as internal reference controls (25). However, most previous studies have focused on differential expression, biological mechanisms, or prognostic associations. The clinical question addressed in the present study was distinct: whether EV-associated snoRNA measurements could add diagnostic information beyond routinely measured serum tumor markers used in NSCLC assessment.

All four candidate snoRNAs showed lower ΔCt values in NSCLC samples, indicating higher relative abundance in the small-EV-enriched fraction. The biological mechanisms underlying these differences remain uncertain. Altered transcriptional regulation, selective RNA packaging into extracellular vesicles, changes in ribosome-related pathways, and differences in cellular sources of circulating vesicular material may contribute to the observed patterns (26–28).

CEA and CYFRA21–1 were selected as the clinical reference model because both markers are routinely used in lung cancer evaluation, although their sensitivity is limited when applied individually and their concentrations may increase in nonmalignant pulmonary conditions (18, 29). Previous studies have also shown that serum marker combinations may achieve moderate discrimination but remain insufficient for early-stage disease detection (30).

In the present cohort, incorporation of the four snoRNAs increased sensitivity by 17.5 percentage points compared with the clinical model while maintaining high specificity. This pattern suggests that EV-associated snoRNA signals may capture molecular information not fully represented by conventional serum markers. However, diagnostic thresholds were derived internally and require prospective evaluation. The six-marker model retained discrimination in early-stage analyses, including stage 0-I, stage 0-II, and stage I disease. These findings provide a rationale for further investigation in clinically relevant populations. Nevertheless, the current analyses compared NSCLC cases with healthy controls rather than individuals undergoing evaluation for pulmonary nodules or other benign lung conditions. Whether the model improves diagnostic decision-making in real-world pathways remains unknown.

Several limitations should be considered. First, the retrospective single-center case-control design and the use of healthy controls may have resulted in overestimation of diagnostic performance. Validation in clinically representative populations, including patients with benign pulmonary diseases and indeterminate pulmonary nodules, is required. Second, external validation was unavailable. Although internal cross-validation and calibration analyses reduced optimism within the source cohort, model performance requires confirmation across independent populations, institutions, and analytical workflows. Third, the discovery cohort was small, and none of the candidate snoRNAs remained significant after false-discovery-rate correction. Therefore, the four-snoRNA panel should be considered exploratory and requires confirmation using predefined analytical criteria in larger cohorts. Fourth, age imbalance between cases and controls may have introduced residual confounding. Although the model maintained discrimination after age- and sex-matched analysis, unmeasured confounding cannot be excluded. Finally, EV characterization and qRT-PCR analytical validation remain incomplete. The current data support an EV-enriched fraction rather than purified exosomes, and further assessment of EV purity, assay performance, and reference-gene stability is needed before clinical translation.

## 5 Conclusions

A serum small-EV-associated four-snoRNA panel provided additional diagnostic information when combined with CEA and CYFRA21–1 in this exploratory NSCLC case-control study. The combined six-marker model showed consistent discrimination during repeated internal cross-validation and in analyses restricted to early-stage disease and age/sex-matched cohorts. However, these findings remain preliminary. Prospective multicenter studies incorporating clinically representative control populations and standardized analytical workflows are needed to determine the clinical utility of this panel.

## Data Availability

The original contributions presented in the study are included in the article/supplementary material. Further inquiries can be directed to the corresponding author.
